# Conjugated PNC-27 peptide/PEI-superparamagnetic iron oxide nanoparticles (SPIONs) as a double targeting agent for early cancer diagnosis: *In vitro* study

**DOI:** 10.22038/IJBMS.2022.65590.14430

**Published:** 2022-10

**Authors:** Reihaneh Rahmani, Majid Darroudi, Mohsen Gharanfoli, Jamshidkhan Chamani, Mehran Gholamin, Maryam Hashemi

**Affiliations:** 1 Department of Laboratory Sciences, School of Paramedical Sciences, Mashhad University of Medical Sciences, Mashhad, Iran; 2 Research Institute of Applied Sciences (ACECR), Shahid Beheshti University, Tehran, Iran; 3 Nuclear Medicine Research Center, Mashhad University of Medical Sciences, Mashhad, Iran; 4 Department of Medical Biotechnology and Nanotechnology, School of Medicine, Mashhad University of Medical Sciences, Mashhad, Iran; 5 Department of Cell and Molecular Biology, Faculty of Basic Sciences and Advanced Technologies in Biology, University of Science and Culture, Tehran, Iran; 6 Department of Biology, Faculty of Sciences, Mashhad Branch, Islamic Azad University, Mashhad, Iran; 7 Immunology Research Center, Mashhad University of Medical Sciences, Mashhad, Iran; 8 Department of Pharmaceutical Biotechnology, School of Pharmacy, Mashhad University of Medical Sciences, Mashhad, Iran; 9 Nanotechnology Research Center, Pharmaceutical Technology Institute, Mashhad University of Medical Sciences, Mashhad, Iran

**Keywords:** B-PEI, Cytotoxicity effect, Iron oxide, PNC-27 peptide, SPIONs, Targeted cancer diagnostic

## Abstract

**Objective(s):**

Superparamagnetic iron oxide nanoparticles (SPIONs) have been considered promising non-invasive imaging tools in medicine. However, their high surface energy leads to NPs aggregation, while non-targeted SPIONs can cause cytotoxic effects on normal cells. In this work, we evaluated the *in vitro* potential of polyethyleneimine (PEI)-SPIONs targeted by PNC-27 peptide as a double targeting agent throughout early cancer diagnosis.

**Materials and Methods:**

Initially, PEI was conjugated to PNC-27 with HDM-2-binding domain. Then, SPIONs were loaded into PEI-PNC-27 through the ligand exchange method. The physicochemical characteristics of the synthesized NPs were evaluated. The cytotoxicity and targeting efficiency were assayed against HT-29 and CT-26 cell lines along with NIH-3t3 as normal cells by MTT method and Prussian blue staining test, respectively.

**Results:**

The mean diameter of synthesized carriers was obtained in the range of 86.6 – 116.1 nm with a positive charge. According to the cytotoxicity results, the binding and uptake abilities of the PNC-27 peptide by cancer cells were significantly higher than that of the NIH-3t3 cells. However, the results were indicative of the more toxic impacts of targeted synthesized NPs against CT-26 cancer cell line when being compared with HT-29 cells, which may be caused by the different cytotoxicity mechanisms of NPs. In addition, the targeted carriers and SPIONs were present inside and around the cells with HDM-2 expression along with only a few non-targeted vectors, while displaying no appearance throughout the normal cell.

**Conclusion:**

The results indicated the efficiency of targeted PEI-coated SPIONs for cancer diagnostic applications.

## Introduction

Cancer stands as a leading cause of morbidity and mortality in the world. However, early diagnosis of this disease can increase the chances of survival and efficiency of treatments. The current diagnostic imaging techniques applied in cancer clinical management include magnetic resonance imaging (MRI) and X-ray computed tomography (CT) (1), positron emission tomography (PET), and single-photon emission tomography (SPECT) (2, 3). However, these methods can only detect proliferated and metastasized cancer cells with significant changes. Recently, nanotechnology attracted the attention of many for performing the early detection of cancer mainly due to providing a large surface area to volume ratio that can be covered with cancer-targeting agents. As a promising non-invasive imaging method, magnetic NPs possess the properties of magnetic and NPs at the same time (4). Superparamagnetic iron oxide NPs (SPIONs) are some of the most commonly used magnetic NPs with unique properties such as biocompatibility, simple synthesis, magnetic superchargers, and small size while offering the possibility of surface modification (5-8). The wide applications of this material can be observed in various therapeutic applications such as MRI, targeted delivery of drugs or genes, hyperthermia, and tissue engineering (9-11). Although SPIONs are sized in the range of 5–100 nm, their high surface energy typically leads to the aggregation of NPs. One of the approaches for reducing the intrinsic magnetism and stabilizing aqueous SPIONs is the coating of NPs with specific polymers such as poly (lactic-co-glycolic acid)  (PLGA) (12), Polyethylene glycol (PEG) (13), Chitosan (14), and Polyethyleneimine (PEI) (15, 16). The complex of high cationic charge density of PEI with drugs or genes can improve their pharmacokinetic properties and cellular uptake. Recently, our group reported a novel green co-precipitation method for synthesis of SPIONs at laboratory temperature and air atmosphere with the application of a few chemical solutions, which proved to be much easier than the usual chemical co-precipitation method (17, 18). 

In addition, we exerted quince seeds extract as a stabilizer agent to decrease the accumulation of SPIONs (17). Recently, targeted SPIONs were enhanced to identify specific cell-surface receptors on cancer cell types and reduce their cytotoxicity. As a protein receptor, HDM-2 is generally over-expressed on the surface of many cancer cells while having a minimal presence on the membranes of normal cell lines. Peptide PNC-27 with the self-identifying sequence of H-Pro-Pro-Leu-Ser-Gln-Glu-Thr-Phe-Ser-Asp-Leu-Trp-Lys-Leu-Leu contains the HDM-2-binding domain, which is located near the plasma membrane. The linking of the MRP segment (a transmembrane penetrating peptide) to the PNC-27 peptide induces pore-forming activity in the cell membrane and leads to cell necrosis (19). In this study, we evaluated the *in vitro* potential of targeted PEI-SPIONs by PNC-27 peptide without MRP domain as a double targeting agent to be considered for the early diagnosis of cancer.

## Materials and Methods

***Materials***

Branched-Polyethylenimine (B-PEI~10 kDa) is a highly branched liquid water-soluble polyamine with high cationic charge density which was purchased from Sigma Aldrich (Germany), N-Succinimidyl 3-(-pyridine-2-thione). Propionate (SPDP) was obtained from Fluka (Buchs, Switzerland). PNC-27 peptide, Ac-CGGGPPLSQETFSDLWKLL, was acquired from Chinapeptides Co. (Shanghai, China) and >97% purified by HPLC and mass spectrographic methods. Iron Stain Kit (Prussian blue Stain, USA). All of the chemicals and solvents were of analytical grade and water was deionized before being utilized throughout the experiments.

***Green synthesis of SPIONs***

**Scheme 1:**
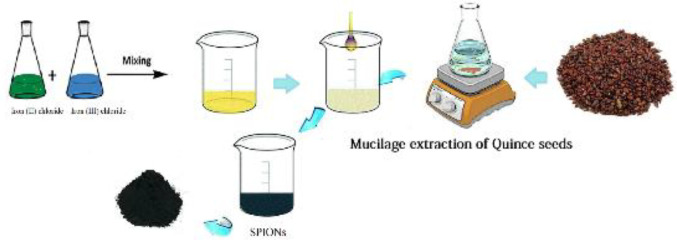
Schematic diagram of green synthesis of SPIONs by quince seed mucilage

The SPIONs were synthesized in accordance with our previous report’s description (17). Briefly, iron (II) chloride tetrahydrate and iron (III) chloride with a molar ratio of 2:1 were added to 150 ml of quince seeds extract and stirred continuously at 300 rpm at a temperature of 60 ^°^C. After 30 min, the pH of the solution was adjusted to 11 by the usage of NaOH (0.20 M) solution. The obtained NPs were collected by a magnet and centrifuged (9,000 rpm, at 4 ^°^C for 20 min), and the resultant was washed several times with water. Finally, the produced SPIONs were dried in an oven (70 °C) for 24 hr (Scheme 1).

***Coated of SPIONs using B-PEI***

The preparation of loaded SPIONs was done through a ligand-exchange method with the usage of B-PEI (20, 21). To state in detail, SPIONs (1.0 mg) were dispersed into B-PEI solution (10 mg in 1.0 ml of chloroform) by the utilization of a sonicating bath for 3 min. Then, the reaction mixture was stirred overnight at room temperature. Once the SPIONs-Loaded B-PEI was precipitated by the addition of 10 ml of hexane, the resultant was collected through centrifugation (8,000 rpm, at 20 ^°^C for 20 min) and washed twice with hexane; then the product was vacuum-dried to remove the organic solvent. Subsequent to dispersing the product into double-distilled water under sonication, it was filtered through a 220 nm membrane for removing the large aggregates and stored at -20 ^°^C for the upcoming experiments. The amount of PEI in the final product was determined by means of 2,4,6-trinitrobenzene sulfonic acid (TNBS) assay as described previously (22). 

***Synthesize of 3-(2-pyridyldithiol) propionate modified B-PEI***

B-PEI was conjugated to the hetero-bifunctional cross-linker N-succinimidyl 3-(-pyridine-2-thione) propionate (SPDP).

 For this purpose, 0.50 ml of B-PEI stock 10 kDa (10.0 mg/ml in distilled water, pH= 7.2) was added to a 1.50 ml PBS buffer solution. Then, dissolved SPDP in DMSO (1.0 mg and 2.0 mg for 5 and 10% surface amine substitution, respectively) was appended drop wisely to B-PEI solution and stirred for 18 hr at room temperature to be purified through the application of dialysis tubing (Amicon, Beverly, MA, USA, molecular weight cut-off 3 kDa). Thereafter, the ultimate products (PEI-SPDP 5% and PEI-SPDP 10%) were stored at 4 ^°^C. The degree of SPDP conjugation was determined by measuring the absorption of 3-(2-pyridyldithiol) propionate (PDP) and utilizing the excess of DTT at 343 nm with the molar absorptivity of 8.08×10^3^ M^-1^ cm^-1^.

***Conjugation of PNC-27 peptide to PEI-SPDP***

Peptide conjugation was performed according to the disulfide bond of PEI-SPDP and PNC-27; one mole of SPDP linker must react with one mole of a peptide (23). PEI-SPDP (5.0 mg of PEI) containing 1.25 µmol and 2.7 µmol of SPDP was mixed with 2.5 mg or 5.5 mg of peptide (two-fold excess) for 5% and 10% substitution, respectively; then, they were incubated for 4 hr at room temperature. To measure the amount of peptide conjugation, the absorption of released thiopyridone was determined at 343 nm. As the last step, the unreacted peptides were removed by dialysis against distilled water through centrifugation, which involved the application of an Amicon® ultra centrifugal filter (6,000 rpm, at 4 ^°^C for 15 min). The purified products (PEI-SPDP %5-PNC-27 and PEI-SPDP %10-PNC-27) were kept frozen at -20 ^°^C until further usage. 

***Coated of SPIONs by PEI-SPDP-PNC-27***

According to the mentioned ligand exchange method, PEI-SPDP 5%-PNC-27 (containing 1.0 mg of PEI and 225 µl of PNC-27) was added to 1.0 mg/ml of SPION solution. Also, PEI-SPDP 10%-PNC-27 (containing 1.5 mg of PEI and 270 µl of PNC-27) was added to 1.5 mg/ml of SPION solution. Then, both of the mixtures were stirred at room temperature for 3 hr by the use of a magnet to have the reaction mixture stirred overnight at room temperature.

The loaded SPIONs in PEI-SPDP-PNC-27 were precipitated by adding 10 ml of hexane, which were then collected through centrifugation (6,000 rpm, at 4 ^°^C for 15 min). All of the products were washed twice with hexane and vacuum-dried to remove the organic solvent; in this way, they were dispersed into double-distilled water under sonication and filtered through a 220 nm membrane (to remove large aggregates and unreacted solvents), and after that to be kept at -20 ^°^C for further usage. The amount of PEI in the final product was determined by the means of 2,4,6-trinitrobenzene sulfonic acid (TNBS) assay as described previously (22). The abbreviations of synthesized formulations are presented in Table 1.

***Physicochemical characterization of synthesized NPs***

*Particle size and zeta potential measurements*

The hydrodynamic diameters and zeta potential of SPIONs and obtained nano-vectors were measured through Dynamic Light Scattering and Laser Doppler Velocimetry, respectively, by application of a Malvern Nano ZS instrument and DTS software (Malvern Nano ZS, UK). For this purpose, 1.0 mg of synthesized NPs was suspended in 1.0 ml of double‐distilled water (24) and sonicated for 20 min to turn into a homogeneous suspension. The gathered data represent the average ±standard deviations, while the analysis was performed in triplicate and all of the measurements were recorded at 25 ^°^C.

*Morphological studies*

The morphological study of SPIONs, PS 5%-PNC@SPI, and PS 10%-PNC@SPI was characterized by utilization of a Field Emission Scanning Electron Microscope (FE-SEM; TESCAN Mira3, Czech Republic), which operated at 5.0 kV accelerating voltage and 1.5 cm working distance between the samples and detector.

*Determination of iron concentration*

Atomic Absorption Spectroscopy (AAS) (Varian, 240FS FLAME AA Spectrometer, USA) was used to determine the concentration of Fe_3_O_4_ NPs in P@SPI, PS 5%-PNC@SPI, and PS 10%-PNC@SPI (25). Initially, a calibration diagram was obtained using standard solutions within the concentration range of 1.25, 2.50, 5.0, and 10.0 µg/ml. Then, the absorption of the unknown solution was measured and the concentration of the unknown element was determined from the standard curve.

*Evaluation of integrity of PNC-27 peptide*

The performance of Tricine-Sodium Dodecyl Sulfate Polyacrylamide Gel Electrophoresis (SDS PAGE) was considered to confirm the conjugation of PNC-27 peptide to PEI, which was in accordance with Laemmli with some modifications (26). Briefly, PS 5%-PNC@SPI and PS 10%-PNC@SPI were analyzed by the usage of 15.0 % acrylamide separation gels with a 5.0 % stacking gel under reducing conditions. Samples were diluted at a 1:1 ratio with sample buffer and boiled at 95 ^°^C (27). Subsequent to the loading of samples and electrophoresis (80 v, 30 mA for 30 min), the gels were stained with Coomassie brilliant blue 0.1% w/v.

***In vitro*** ***cellular study***

*Cell culture*

Two cancer cell lines that overexpress HDM-2, including HT-29 cell line (human colorectal adenocarcinoma cell line) and CT-26 cell line (murine colorectal carcinoma cell line)), as positive cells were procured from ATCC along with NIH-3t3 (mouse embryonic fibroblast cell line) as a negative cell to function like the normal cells. The HT-29 and CT-26 cell lines were cultured in RPMI (Roswell Park Memorial Institute 1640), while the NIH-3t3 cell line was grown in DMEM (Dulbecco’s Modified Eagle’s medium from Biosera, UK).

Meanwhile, both of the culture mediums were supplemented with 10.0% fetal bovine serum (VWR, Visalia, CA, USA) and 1.0% penicillin-streptomycin (Sigma, St. Louis, MO, USA). The cells were maintained in an incubator at 37 ^°^C with a humidified atmosphere of 5.0% CO_2_. The cells that were in the exponential growth phase were exerted to perform all of the experiments.

*Cytotoxicity assay*

A comparison experiment was done to determine the targeting and cytotoxicity qualities of SPIONs, PEI, and nano-vectors containing both SPIONs and PEI, which was executed through the usage of 3-(4,5-Dimethylthiazol-2-yl)-2, 5-diphenyltetrazolium bromide (MTT assay) (28). The cell lines (HT-29, CT-26, and NIH-3t3) were seeded in a 96-well plate at the density of 1×10^4^ cells/well in the assigned medium and incubated at 37 ^°^C. After 24 hr, the cells were treated with a serial concentration of SPIONs NPs (0.012 to 3.2 µg/ml), PEI (0.15 to 80.0 µg/ml), P@SPI, PS 5%-PNC@SPI, and PS 10%-PNC@SPI (containing 0.02 to 0.8 µg/ml SPIONs) to be incubated for 24 hr. Subsequently, the medium was aspirated to have the wells cleansed with PBS and the procedure was followed by appending 200.0 μl of fresh medium to each well. Then, 10.0 µl of MTT (5.0 mg/ml in PBS) solution was added into each well and after 4 hr of incubation, the total medium was replaced with DMSO (100 µl). The absorbance of samples was recorded at 570 nm through the application of a BioRad microplate reader with a reference wavelength of 630 nm. The cell viability (%) relative to control wells containing cell culture medium without treatment was calculated using A_test_ / A_control_ ×100.

*Qualitative binding study*

The existing intracellular iron can be detected through the method of Perl’s Prussian Blue (PB) staining (29). Briefly, CT-26 and HT-29 cancer cells were seeded in six-well plates at the density of 2.0 ×10^5^ cells/well and incubated overnight.

 Then, the cells were washed and exposed to the samples of SPIONs (2.0 µg/ml), P@SPI, PS 5%-PNC@SPI, and PS 10%-PNC@SPI containing 3.4, 3.2, and 2.4 µg/ml SPIONs, respectively to be incubated afterward for 4 hr. Then, the cells were washed three times with cold PBS and fixed with 4.0% v/v formaldehyde for 10 min to be incubated for 20 min with 4.0% potassium ferrocyanide and 4.0% HCl solution. Subsequently, the cells were cleaned again with PBS to be stained with a nuclear fast red solution for 30 min. Finally, the cells were washed with DI water and rapidly dehydrated through alcohol to go under surveillance by the usage of a Zeiss Axiovert 40 CFL microscope. As the control sample, NIH-3t3 cells (normal cell line) were treated with a similar procedure.

***Statistical analysis***

All of the statistical analyses were performed through the utilization of Graph Pad Prism 6 Software. Results were expressed as mean±standard deviation (SD).

## Results

**Figure 1:**
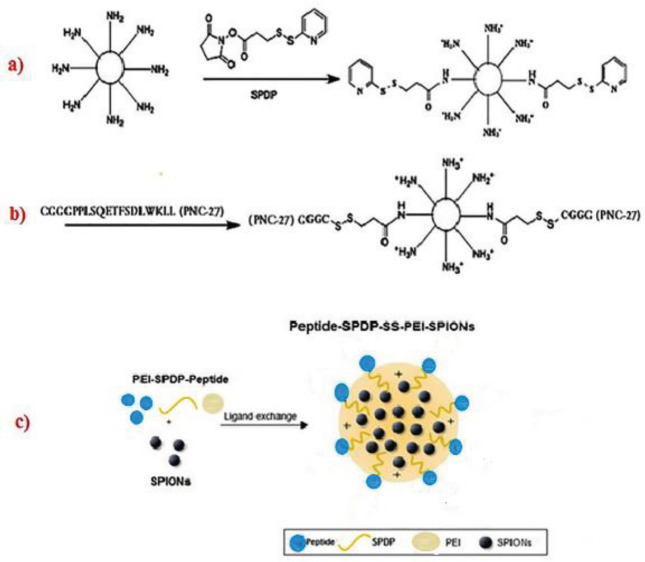
Steps of creating synthesized nano-vectors; Formation of the PEI-B-SS-SPDP compound (a), Conjugation of PNC27 peptide loading to PS (b), Loading of SPIONs in PS-PNC conjugate by ligand exchange method, and creation of PS-PNC@SPI nano-vector (c)

PS: PEI-SPDP; PS-PNC: PEI-SPDP-Peptide; PS-PNC@SPI: PEI-SPDP-Peptide-SPION

The focus of this study was centered on PNC-27 peptide conjugated PEI-SPIONs NPs as a double targeting agent for the early diagnosis of cancer. The schematic representation of the synthesized PS-PNC@SPI nano-vector is presented in Figure 1.

***Preparation of peptide-conjugated PEI***

To develop the targeted SPIONs, PEI was initially activated with SPDP as displayed in Figure 1 (a). Accordingly, about 77% and 86% of primary amine contents of PS 5% and PS 10% were modified with SPDP, respectively. Then, the peptide was added to PEI-DTP in the assigned concentrations to achieve the conjugation of 5 and 10% of primary amines with peptides (Figure 1 (b)).

 The amount of released 2-thiopyridone in the presence of peptide indicated that the yield of the reaction was about 90% for the two formulations. The concentration of PEI in final products in conformity to the TNBS test is provided in Table 2.

***Characterization of synthesized NPs***

The mean diameter of synthesized SPIONs was obtained in the range of 86.6–116.1 nm with a positive charge through application of the DLS method. According to the displayed size (Z-average) and zeta potential of all the NPs in Table 3, the loading of SPIONs into B-PEI or PS-PNC-27 resulted in a significant decrease in these values. However, in this study, all of the obtained NPs contained a suitable size and adequate positive charge for attaching to the surface of cells.

**Figure 2:**
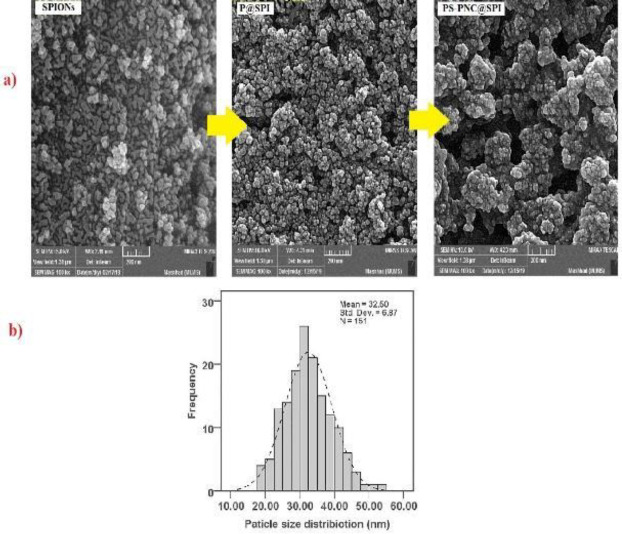
(a) Morphologies of particles. FE-SEM images of SPIONs, P@SPI, and PS-PNC@SPI nano-vector. (b) Obtained particle size of PS-PNC@SPI by ImageJ software

We evaluated the morphology of obtained NPs through the employment of FE-SEM, which was synthesized, and the results were illustrated in Figure 2. Following the captured FE-SEM images, the shape of NPs was detected to be spherical as displayed in Figure 2 (a) within the size range of 20–50 nm which can be seen in Figure 2 (b). The obtained outcomes confirmed the smooth morphology and particle size of the nanoparticle’s distribution plot of PS-PNC@SPI, which was calculated to be approximately 32.50 (Figure 2 (b)). 

***Iron content determination***

The existing iron concentration in final products was determined by atomic absorption spectrophotometry (AAS) at 248 nm (SpectrAA-10 plus, Varian, France) (Table 4).

 A calibration curve was obtained by treating an acidic solution of FeCl_3_ (1.0 g/l) in similar conditions. 

***Tricine-SDS page***

**Figure 3:**
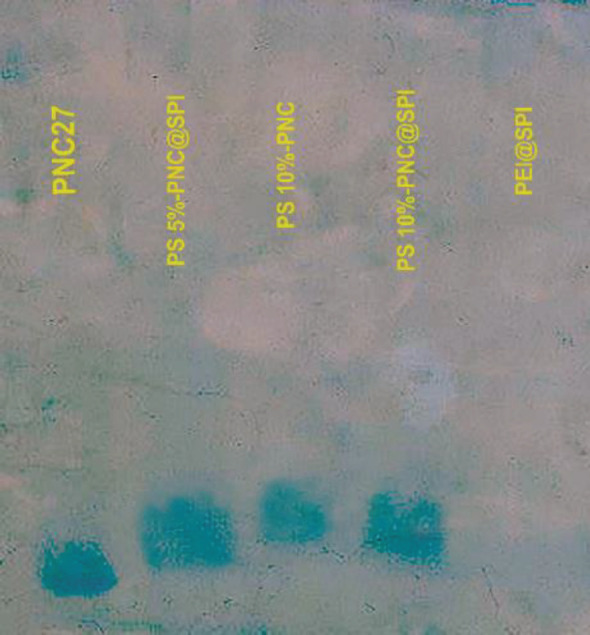
Coomassie-stained SDS-PAGE photograms of PNC27 peptide in PS 5% -PNC@SPI and PS 10%-PNC@SPI

The tricine-SDS-PAGE test was performed to confirm the presence of peptide in PS-PNC@SPI targeted nano-vector and have it compared with non-targeted P@SPI nano-vector. As exhibited in Figure 3, the presence of the PNC-27 peptide in the targeted nano-vector confirmed the binding of the PNC-27 identifier peptide to the B-PEI polymer.

***In vitro cytotoxicity assay***

**Figure 4:**
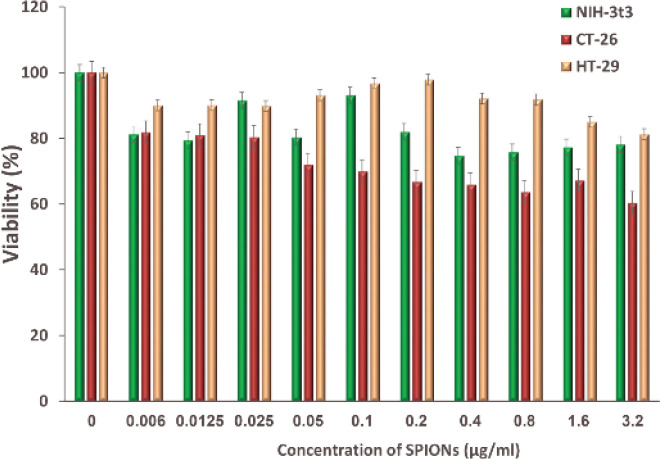
Evaluation of the cytotoxicity effect of SPIONs on HT-29 and CT-26 cancer cell lines and NIH-3t3 normal cell line by MTT assay after 24 hr (Mean±SD; n=5)

The cytotoxicity of SPIONs, B-PEI-10 KDa, P@SPI, PS 5%-PNC@SPI, and PS 10%-PNC@SPI nano-vectors were evaluated against positive cells, HT-29 and CT-26, and also negative cell, NIH-3t3, by the means of MTT assay. In conformity to Figure 4, the highest survival rate against SPIONs was observed in HT-29 cell lines (more than 80%) and the highest effect of toxicity was observed in CT-26 cell line after 24 hr. The cell viability percentage was higher than 60% in three cell lines against all of the studied concentrations of SPIONs.

**Figure 5:**
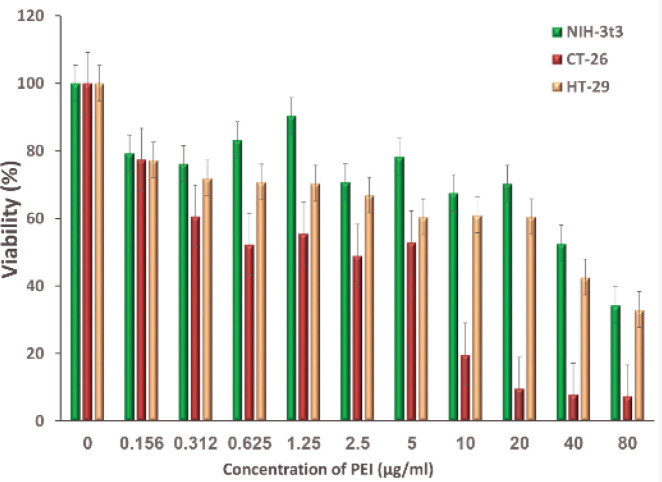
Evaluation of cytotoxicity effect of B-PEI 10 KDa polymer on HT-29 and CT-26 cancer cell lines and NIH-3t3 normal cell line by MTT assay after 24 hr (Mean±SD; n=5)

The toxicity results of B-PEI 10 Ka indicated a survival rate of higher than 60% at concentrations up to 20 µg/ml for NIH-3T3 and HT-29 cell lines (Figure 5). However, the highest toxicity of PEI polymer was obtained in the case of CT-26 cancer cell line, which suggests the higher sensitivity of mouse clone cancer cells.

In this section, we evaluated the cytotoxicity effect of synthesized NPs containing 0.025–0.8 µg/ml of SPIONs. The amount of PEI polymer (determined by TNBS test) and Fe in NPs that contained 0.8 µg/ml of SPIONs (the highest exerted concentration of SPIONs) are demonstrated in Table 5. 

**Figure 6:**
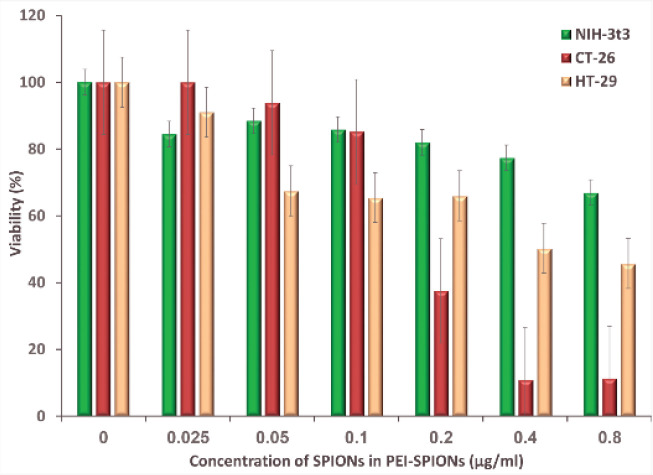
Evaluation of cytotoxicity effect of non-targeted P@SPI nano-vector on HT-29 and CT-26 cancer cell lines and NIH-3t3 normal cell line by MTT assay after 24 hr (Mean±SD; n=5)

According to Figure 6, SPIONs-loaded PEI induced the lowest toxicity in normal cells and also caused 50% of survival in concentrations up to 0.4 µg/ml for the HT-29 cell line. However, the highest toxicity was expected to be observed throughout the CT-26 cell line, since the application of concentrations up to 0.1 µg/ml is typically required to achieve a cell survival of more than 50% in the case of non-targeted P@SPI nano-vector.

**Figure 7:**
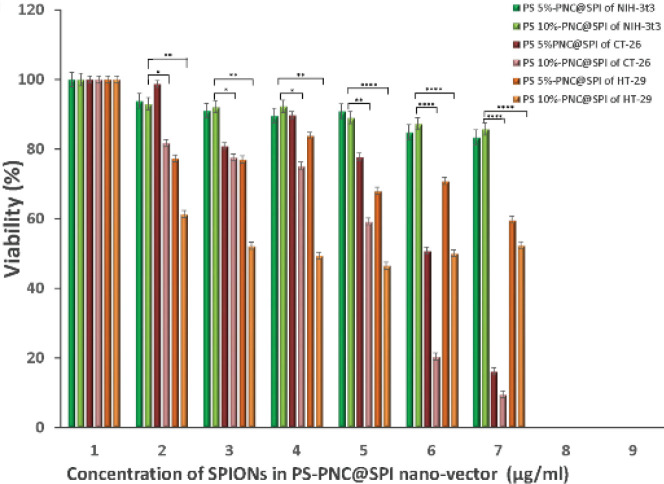
Evaluation of cytotoxicity effect of PS 5%-PNC@SPI and PS 10%-PNC@SPI nano-vector on HT-29 and CT-26 cancer cell lines and NIH-3t3 normal cell line by MTT assay after 24 hr (Mean±SD; n=5)

The cytotoxicity results of targeted NPs indicated more than 50% of cell survival for both cases of NIH-3t3 normal and HT-29 cancer cell lines throughout all of the experimented concentrations (Figure 7). Furthermore, PS 10%-PNC@SPI displayed higher cytotoxicity than PS 5%-PNC@SP. In addition, synthesized carriers showed a significant toxicity effect against CT-26 cell line at a concentration of more than 0.2 µg/ml of SPIONs.

***Prussian blue staining***

Prussian Blue staining (PB) was used to confirm the uptake of SPIONs in cells (30). For this purpose, the cells were treated with non-targeted and targeted NPs that contained 0.2 and 0.1 µg/ml of SPIONs (non-toxic concentrations), respectively, for 2 hr.

**Figure 8:**
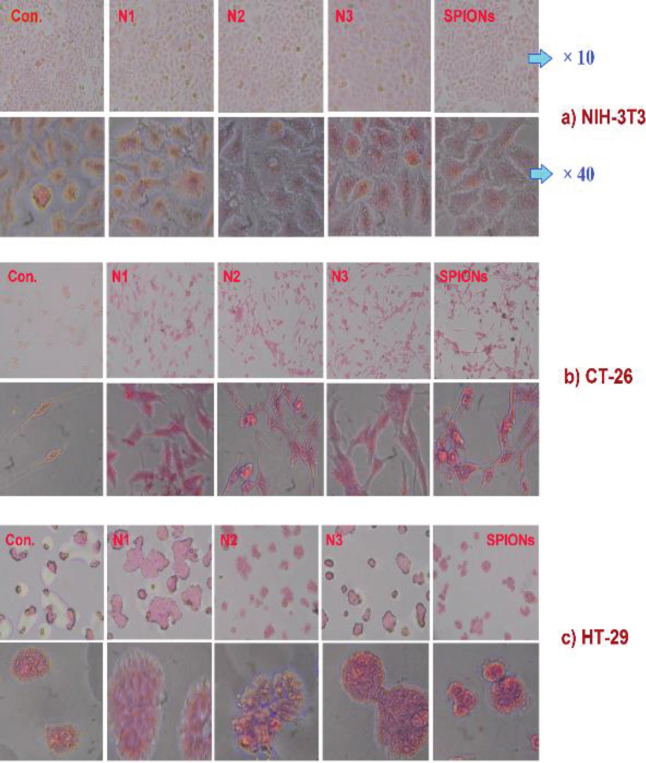
Confirmation of SPIONs and their derivatives around the cell membrane in CT-26 (b) and HT-29 (c) cancer cell lines relative to the normal NIH-3T3 (a) cell. (Control cells (Con.), Non-targeted nano-vector P@SPI (N1), targeted nano-vectors PS 5%-PNC@SPI (N2) and PS 10%-PNC@SPI (N3), and SPIONs)

 As exhibited in Figure 8, Dyed SPIONs formed around the cancer cell membranes in targeted nano-vectors, which confirms the binding of peptide PNC-27. Also, it confirmed the binding of PS 5%-PNC@SPI and PS 10%-PNC@SPI targeted nano-vectors to the surface of cancer cells by HDM-2 marker. This test was performed against normal NIH-3T3 cells as well. The absence of SPIONs around these cells confirmed the binding of targeted PNC-27 to the HDM-2 receptor on the surface of human and mouse clone cancer cells.

**Table 1:** List of abbreviations of synthesized nano-vectors

**Formulations**	**Abbreviated name of vectors**
PEI-s	P@SPI
PEI-SPDP 5%	PS 5%
PEI-SPDP 10 %	PS 10%
PEI-SPDP 5%-PNC27	PS 5%-PNC
PEI-SPDP 10%-PNC27	PS 10%-PNC
PEI-SPDP 5%-PNC27@SPIONs	PS 5%-PNC@SPI
PEI-SPDP 10%-PNC27@SPIONs (PEI-SPDP-PNC27@SPIONs)	PS 10%-PNC@SPI (PS-PNC@SPI)

PEI: Polyethyleneimine; SPDP: N-Succinimidyl 3-(-pyridine-2-thione) Propionate; SPION: Superparamagnetic iron oxide NPs

**Table 2:** Concentration of PEI polymer in synthesized products

**Sample**	**P@SPI**	**PS 5%-** **PNC**	**PS 10%-** **PNC**	**PS 5%-** **PNC@SPI**	**PS 10%-** **PNC@SPI**
**PEI (µg/100 µl)**	65.81	72.30	11.38	30.54	68.61

PEI: Polyethyleneimine; P@ SPI: PEI-SPION; PS 5%-PNC: PEI-SPDP-5% Peptide; PS 10%-PNC: PEI-SPDP-10% Peptide; PS 5%-PNC@SPI: PEI-SPDP-5% Peptide-SPION; PS 10% -P NC@SPI: PEI-SPDP-10% Peptide-SPION

**Table 3:** Size, PDI, and zeta potential of obtained NPs by DLS

**ξ-potentials(mV)** **± SD**	PDI*	**Z**_AVE_ ** (nm)** **± SD**	**NPs**
62.9 ± 2.1	0.16	145.5 ± 6.2	**SPIONs**
61.7 ± 1.3	0.11	133.8 ± 3.6	**P@SPI**
30.2 ± 3.2	0.11	102.1 ± 2.1	**PS 5%-PNC@SPI**
25.1 ± 3.4	0.38	96.2 ± 2.3	PS 10%-PNC@SPI*

PDI: Particle size dispersion; DLS: Dynamic Light Scattering

P@ SPI: PEI-SPION; PS-PNC@SPI: PEI-SPDP-Peptide-SPION

**Table 4:** Amount of iron in each product by atomic absorption spectroscopy (AAS)

**NPs**	**Fe (** **µ** **g/ml)**
**P@SPI**	56 ± 5.1
**PS 5%-PNC@SPI**	64 ± 3.0
**PS 10%-PNC@SPI**	80 ± 2.4

P@SPI: PEI-SPION; PS 5%-PNC@SPI: PEI-SPDP-5% Peptide-SPION; PS 10% -PNC@SPI: PEI-SPDP-10% Peptide-SPION

PS 5%-PNC@SPI: PEI-SPDP-5% Peptide-SPION; PS 10% -PNC@SPI: PEI-SPDP-10% Peptide-SPION

**Table 5:** Amount of SPIONs and PEI polymer in nano-vectors at the highest concentration of SPIONs

**PEI** **(µg/ml)**	**Volume of culture medium**	**Amount of sample**	**SPIONs** **(µg/ml)**			**Fe (µg/ml)**	**Sample**
9.3	986 µl	14.2 µl NPs		0.8	56 ± 5.1		**P@SPI**
3.9	987 µl	12.9 µl NPs		0.8	64 ± 3.0		**PS-PNC 5%@SPI**
6.8	990 µl	10 µl NPs		0.8	80 ± 2.4		**PS-PNC 10%@SPI**

P@ SPI: PEI-SPION; PS 5%-PNC@SPI: PEI-SPDP-5% Peptide-SPION; PS 10%-PNC@SPI: PEI-SPDP-10% Peptide-SPION; SPION: Superparamagnetic iron oxide NPs; PEI: Polyethyleneimine

P@SPI: PEI-SPION; PS 5%-PNC@SPI: PEI-SPDP-5% Peptide-SPION; PS 10% -P NC@SPI: PEI-SPDP-10% Peptide-SPION

P@ SPI: PEI-SPION; PS 5%-PNC@SPI: PEI-SPDP-5% Peptide-SPION; PS 10%-PNC@SPI: PEI-SPDP-10% Peptide-SPION

## Discussion

SPIONs stand as potential candidates for cancer diagnosis due to their being biodegradable, biocompatible, and readily endocytosed by a variety of different cell properties. However, the gathered data are indicative of non-targeted SPIONs cytotoxicity towards normal cells. Furthermore, the surface functionalization of SPIONs through various bioligands can increase its specificity to tumor targeting. *Vu-*Quang *et al.* developed an imaging nano-system based on SPIONs that was coated with Pluronic F127-Folateto target folate receptor-expressing cancer cells. According to their *in vitro *results and *in vivo* MRI images, these stable NPs could preferentially target oral squamous cancer (KB) cells by folate receptors expressed on their membranes and through the enhanced negative contrast in tumor-bearing mice (31). In another study, Hu* et al.* reported the high efficiency of arginine-glycine-aspartic acid (RGD) peptide-targeted iron oxide (Fe_3_O_4_) NPs with ultrahigh relaxivity for *in vivo *MR imaging. In this study, Fe_3_O_4_ NPs were coated with PEI by the application of a mild reduction method, which was followed by conjugation with fluorescein isothiocyanate (FI), PEGylated RGD (PEG-RGD), and lastly acetylation of the remaining PEI surface amines.

 The RGD-mediated targeting specificity of NPs to αvβ3 integrin-overexpressing cancer cells was also discovered. It was indicated that these targeted NPs could efficiently target U87MG cells overexpressing αvβ3 integrin with ultrahigh R_2_ relaxivity for MR imaging of tumors (32). In conformity to further examinations, surface functionalization of SPIONs by different bioligands such as dextran, hybrid chitosan-dextran, and protein Hsp70 can increase the specificity of brain tumor targeting (33-35). In addition, SPIONs were conjugated with recombinant human epidermal growth factor (SPION-EGF), and their efficiency in performing the MIR of malignant brain tumors overexpressing EGF receptors was evaluated. The results revealed that these targeted magnetic NPs are capable of displaying high MR contrast in the C6 rat glioma model (36). Recent studies indicated the overexpression of HDM-2 protein on the surface of most cancer cells such as breast, colon, glioblastoma, prostate, and pancreatic cancers, which also prompts the transmembrane pore arrangement in malignancy without causing any impact on normal cells (37, 38). PNC-27 is a membrane-active peptide that contains a corresponding HDM-2-binding domain to the residues of p53 and a transmembrane-penetrating domain (37). In recent years, several studies were focused on the functionality of PNC-27 peptide as a targeting or anticancer agent (37, 39). Darban* et al.* evaluated the targeting ability of conjugated Doxil (a stealth liposomal formulation of doxorubicin) with PNC-27 peptide in CT-26 colon carcinoma cells (HDM2 positive). Their outcomes were suggestive of an exceptionally significant improvement in the restricting and uptake of targeted formulations, which is in contrast with the results of non-targeted Doxil in CT-26 tumor-bearing mice (40). Mokhtarzadeh* et al.* studied the efficiency of PEI 10 kDa and alkylcarboxylated PEI 10 kDa targeted with PNC-27 and PNC-28 peptides involved in interactions with HDM-2 situated for delivering GFP-containing Sure Silencing shRNA plasmid into MCF-7 cells. According to the gathered results, the conjugation of PNC peptides can enhance the gene delivery efficiency of both PEI and alkylated PEI with high-targeted activity exclusively in cancer cells (41). Notwithstanding, perceptions were predictable with the outcomes from a 2D NMR concentrating on the design of PNC-27 appearance that this peptide adopts a strongly amphipathic alpha-helix-loop-alpha-helix structure (19, 42), In our previous work, we performed the synthesis of SPIONs through an eco-friendly and green method by the usage of Quince seeds *(Cydonia oblonga *Miller*) *extract as a bio-stabilizer. That experiment resulted in achieving SPIONs with a spherical shape and a size of less than 50 nm. In addition, *in vitro* cytotoxicity studies indicated the low toxicity effect of the obtained product against A549 cells in concentrations lower than 100 μg/ml (17). In this study, peptide-identifying sequences of PNC-27 were used to target overexpressed HDM-2 on cancer cells. Therefore, we evaluated the *in vitr*o functionality of PNC-27 peptide conjugated PEI-SPIONs as a double targeting agent for the early diagnosis of cancer. As a strong cationic polymer, PEI was exerted to improve the stability of SPIONs. In this regard, PEI with a molecular weight of 10 KD was selected due to its lower cytotoxicity and higher efficiency when compared with the higher and lower Mw of PEI, respectively. In different studies, PEI was used along with SPIONs as a targeted drug or gene delivery system throughout the diagnosis and treatment of cancer (43-45). The diameter and surface charge of NPs affect their uptake, stability, and transfer efficiency (46). Additionally, the results of the current study indicated that the loading of SPIONs into PEI or PS-PNC-27 caused a size reduction, which may be due to the active surface of naked SPION NPs that leads to their tendency for forming aggregations with each other. However, the stabilization of SPIONs that was accompanied by PEI decreased the aggregation percentage of NPs. The lower surface charge of PEI and PNC-peptide (47) compared with SPION NPs may be the reason for the reduced zeta potential of coated SPIONs with PEI or PEI-PNC-27. In the next step, the cytotoxicity of the obtained products was examined by being applied to cancer cells (HT-29 and CT-26). 

As exhibited in Figure 7, the binding and uptake abilities of PNC-27 by HT-29 and CT-26 cells were significantly higher than those of the NIH-3t3 cells due to the high-level expression of HDM2 in HT-29 and CT-26 cell lines resulting in the high uptake of the nanocomplex by the receptor into the cells. Furthermore, the results were indicative of the more toxic impacts of targeted synthesized NPs against the CT-26 cancer cell line when being compared with HT-29 cells, which may be caused by the different cytotoxicity mechanisms of NPs in varying cell lines. Also, PS 5%-PNC@SPI displayed lower cytotoxicity than PS 10%-PNC@SPI in the positive cell lines. To confirm the uptake of NPs in cells, Prussian blue (PB) was performed as a staining method, since it provides data on the accumulation of SPIONs or iron in cells by forming a complex with potassium ferricyanide. According to observations, SPIONs appeared in blue while cell bodies were colored in pink. As it can be perceived more specifically in Figure 8, the targeted nano-vectors (PS 5%-PNC@SPI and PS 10%-PNC@SPI) and SPIONs were present inside and around the cells with HDM-2 expression (HT-29 and CT-26) along with only a few non-targeted nano-vectors (P@SPI), while displaying zero appearance throughout the normal cell (NIH-3t3). Therefore, the appearance of iron oxide NPs around the cells can suggest the potential application of PS-PNC@SPI in molecular imaging and drug delivery, as well as the targeted identification of cancer cells.

## Conclusion

In this work, we evaluated the *in* *vitro* potential of polyethyleneimine-superparamagnetic iron NPs (PEI-SPIONs) targeted by PNC-27 as a double targeting agent throughout the early cancer diagnosis. The results of the cytotoxicity study indicated that the binding and uptake abilities of PNC-27 by HT-29 and CT-26 cells were significantly higher than those of the NIH-3t3 cells due to the high-level expression of HDM2 in HT-29 and CT-26 cell lines. Therefore, this targeting system may offer a novel approach to cancer diagnosis by reducing the inducement of side effects on normal cells. Moreover, the performed *in vitro* studies with the usage of PB as a staining method revealed significant improvements in binding targeted nano-vectors in HDM-2-overexpressing cancer cells when being compared with non-targeted nano-vector (P@SPI). In this system, the PNC-27 sequence can be exerted for targeting HDM-2 on the surface of cancer cells for an efficient diagnosis and also used for biocomplexity purposes. Overall, the obtained results confidently suggest the potential of PNC-27 peptides for serving as an efficient targeting agent for most cancer cells, as well as recommending the promising stand of targeted nano-vectors as targeted agents for diagnostic applications.

## Authors’ Contributions

RR, MD, MGho, and MH designed the experiments; RR performed experiments and collected data; RR, MD, MGha, JC, and MH discussed the results and strategy; MGho and MH supervised, directed, and managed the study; RR, MD, and MH approved the final version to be published.

## Conflicts of Interest

Authors declare no conflicts of interest. The authors have no other relevant affiliations or financial involvement with any organization or entity with a financial interest in or financial conflict with the subject matter or materials discussed in the manuscript apart from those disclosed.
